# Factor structure and internal consistency of the 12-item General Health Questionnaire (GHQ-12) and the Subjective Vitality Scale (VS), and the relationship between them: a study from France

**DOI:** 10.1186/1477-7525-7-22

**Published:** 2009-03-05

**Authors:** Mareï Salama-Younes, Ali Montazeri, Amany Ismaïl, Charles Roncin

**Affiliations:** 1 Laboratory of Social Psychology, Department of Psychology, Rennes II University, Rennes, France; 2 Iranian Institute for Health Sciences Research, ACECR, Tehran, Iran; 3 Laboratory of Sociology and Anthropology, Departement of Sociology, Rennes II University, France

## Abstract

**Background:**

The objectives of this study were to test the factor structure and internal consistency of the 12-item General Health Questionnaire (GHQ-12) and the Subjective Vitality Scale (VS) in elderly French people, and to test the relationship between these two questionnaires.

**Methods:**

Using a standard 'forward-backward' translation procedure, the English language versions of the two instruments (i.e. the 12-item General Health Questionnaire and the Subjective Vitality Scale) were translated into French. A sample of adults aged 58–72 years then completed both questionnaires. Internal consistency was assessed by Cronbach's alpha coefficient. The factor structures of the two instruments were extracted by confirmatory factor analysis (CFA). Finally, the relationship between the two instruments was assessed by correlation analysis.

**Results:**

In all, 217 elderly adults participated in the study. The mean age of the respondents was 61.7 (SD = 6.2) years. The mean GHQ-12 score was 17.4 (SD = 8.0), and analysis showed satisfactory internal consistency (Cronbach's alpha coefficient = 0.78). The mean VS score was 22.4 (SD = 7.4) and its internal consistency was found to be good (Cronbach's alpha coefficient = 0.83). While CFA showed that the VS was uni-dimensional, analysis for the GHQ-12 demonstrated a good fit not only to the two-factor model (positive vs. negative items) but also to a three-factor model. As expected, there was a strong and significant negative correlation between the GHQ-12 and the VS (r = -0.71, P < 0.001).

**Conclusion:**

The results showed that the French versions of the 12-item General Health Questionnaire (GHQ-12) and the Subjective Vitality Scale (VS) are reliable measures of psychological distress and vitality. They also confirm a significant negative correlation between these two instruments, lending support to their convergent validity in an elderly French population. The findings indicate that both measures have good structural characteristics.

## Background

### The General Health Questionnaire (GHQ)

The General Health Questionnaire (GHQ) was developed in England as a screening instrument to identify psychological distress in primary care settings [1]. It was originally designed as a 60-item instrument but several shortened versions are currently available, including the GHQ-30, the GHQ-28, the GHQ-20 and the GHQ-12. The shortest version of the questionnaire (GHQ-12) has been extensively validated and used in a number of countries and in different languages [2-6]. Since this version is brief, simple and easy to complete, and its application as a screening tool in research settings is well documented, it was decided to translate the GHQ-12 from English into French and to examine its psychometric properties and factor structure (i.e. one, two or three factors) in a sample of elderly French adults.

### The Subjective Vitality Scale (VS)

The Subjective Vitality Scale (VS) is a seven-item instrument that was developed by Ryan and Fredrick to measure vitality [7]. It has two versions: an Individual Difference Level Version, which asks individuals to respond to each item by indicating the degree to which it is generally true in their lives; and the State Level Version, which asks individuals to respond to each item in terms of how they are feeling at that moment [8]. The Individual Difference Level Version was found to relate positively to self-actualisation and self-esteem and negatively to depression and anxiety, while the State Level Version relates negatively to physical pain and positively to the amount of autonomy support in a particular situation [8,9]. Another version of the instrument contains six items. This was developed by Bostic et al. using confirmatory factor analysis; since one of the original seven items was negatively worded, they excluded it to yield a model that fitted their data better [10]. The questionnaire is a brief measure of vitality *pre se *and is simple and easy to complete, and its application as a uni-dimensional instrument in research settings is well documented [7,10]; so it was decided to translate it into French and to examine its factor structure and internal consistency for the same population.

### The relationship between the two instruments

Evidence indicates a strong link between vitality and a variety of health conditions [11]. Thus, as suggested, it may be hypothesized that the vitality score will be lower in cases of somatic pain, physical symptoms and ineffective body functioning [7]. We were therefore interested in testing this hypothesis and examining whether there is a relationship between psychological distress and vitality. The relationship between the GHQ-12 and the VS has not yet been tested.

## Methods

### Translation and data collection

The standard "forward-backward" procedure was applied to translate the questionnaires (the GHQ-12 and the VS) from English into French. Two bilinguals translated the original scales into French. Two independent translators then back-translated the two translated versions into English. The translators were not connected to the study so comparability and meaning equivalence were ensured. Using the different versions, the authors created a provisional French version of each scale. An independent professional revised these provisional versions. In general, minor differences were corrected at this stage by agreement between the different translations and the final versions were made available for this study. Data were then collected from a sample of elderly French adults who practised physical activities regularly in a group. They rated (self-rated) the GHQ-12 and the VS immediately after completing their physical activities.

In Western culture, physical activity is considered a life style model. Many people practise their favourite physical activity, especially after retirement age, in order to be happier and healthier. Since there are associations in France that organise physical activity sessions for older people, we contacted the Rennes association and recruited the sample for this study. The participants practised jogging, walking, cycling, rhythmical gym, yoga, dance and streatching. At the time of the study they were participating at least three times per week for a total of 3–4 hours.

### Measures

#### 1. The General Health Questionnaire (GHQ-12)

This is a widely-used instrument designed to screen for psychological distress. The scale asks whether the respondent has experienced a particular symptom or behaviour recently. Each item is rated on a four-point scale (less than usual, no more than usual, rather more than usual, or much more than usual) and it gives a total score of 12 or 36 on the basis of the scoring method selected. The most common scoring methods are bimodal (0-0-1-1) and Likert scoring (0-1-2-3). Since the latter produces a more acceptable distribution of scores for parametric analysis (less skewed and less kurtosis), we used the Likert scoring style for this study. A higher score indicates a greater degree of psychological distress [1].

#### 2. The Subjective Vitality Scale (VS)

the six-item Subjective Vitality Scale (the Individual Difference Level Version) was used to measure vitality. The scale asks the respondents to indicate the degree to which the six positively-worded statements are true for them in general in their lives. Each item is rated on a 6-point scale (1 = not at all true, 2 = not true, 3 = almost not true, 4 = almost true, 5 = true, 6 = very true). The total score ranges from 6 to 36 with a higher score indicating a better condition [8,10].

### Statistical analysis

Internal consistency was assessed by calculating Cronbach's α coefficient. Values of 0.70 or greater were considered satisfactory [12]. We performed confirmatory factor analyses (CFA) to assess the structures of the two instruments. The intention was to ascertain which model fits the data better. There are different suggestions in the literature about the number, type and cut-off values for goodness-of-fit required for CFA [13,14]. A popular recommendation is to present three or four indices from different areas. Accordingly, we report several goodness-of-fit indicators: the Goodness of Fit Index (GFI), Adjusted Goodness of Fit Index (AGFI), Root Mean Square Error of Approximation (RMSEA) and Relative chi-square (χ^2^/df). The GFI and AGFI are chi-square-based calculations independent of degrees of freedom. The recommended thresholds for acceptable values are ≥ 0.90. The RMSEA tests the fit of the model to the covariance matrix. As a guideline, values of < 0.05 indicate a close fit and values below 0.11 an acceptable fit. The value of χ^2 ^alone may be used as an index, but χ^2 ^divided by the degrees of freedom (χ^2^/df) reduces its sensitivity to sample size (cut-off values: < 2 to 5) [13,14]. Finally, the relationship between the two instruments was tested using the Pearson product moment statistic (Pearson's correlation coefficient = *r*). A significant negative correlation was expected.

### Ethics

The authors informed the subjects about the study objectives, that their participation was voluntary, and they could withdraw at any time. Both oral and written instructions were given to ensure that the items were understood (i.e. that there were no right or wrong answers to the questions and that the participants should freely and honestly state what they think), and the subjects were reassured that their responses were confidential.

## Results

### Descriptive findings

In all, 217 elderly subjects aged 58–72 years (Mean 61.7, SD = 6.2) entered the study. Most of them were female (61%) and most were employed (83%). Using the Likert scale, the mean score was 17.4 (SD = 8.0) for the GHQ-12 (range from 0 to 36), and 22.4 (SD = 7.4) for the VS (range from 6 to 36). These findings indicate that at least 50% of the respondents reported less stress and showed high vitality.

### Factor analysis and internal consistency

#### 1. The General Health Questionnaire (GHQ-12)

(i) The single factor model was used initially but it fitted the data poorly, as shown by the fit indices (Table 1). CFA indicated that a 12-item uni-dimensional model did not fit the data well. We found the following figures: Goodness of Fit Index = 0.72, Adjusted Goodness of Fit Index = 0.63, Root Mean Square Error of Approximation (RMSEA) = 0.2, and χ^2^/df = 8.99.

**Table 1:** The results obtained from confirmatory factor analysis for the GHQ-12 and the VS (n = 217)

**Latent model**	**χ^2^**	**df**	**GFI**	**AGFI**	**RMSEA**	**χ^2^/df**
**GHQ-12**						

One factor	485.26	54	0.72	0.63	0.2	8.99

Two factor	71.96	34	0.93	0.90	0.02	2.11*

Three factor	115.45	51	0.92	0.90	0.03	2.26*

**VS**						

One factor	99.86	9	0.90	0.89	0.2	11.01

GFI: Goodness of Fit Index, AGFI: Adjusted Goodness of Fit Index, RMSEA: the Root Mean Square Error of Approximation.

**Figure 1:**
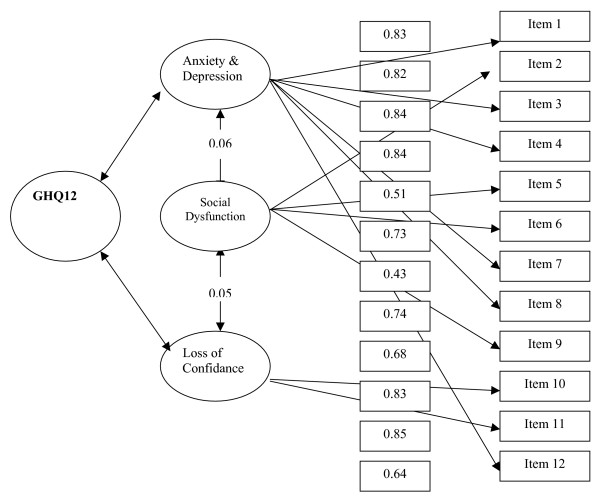
**The results of confirmatory factor analysis of the three-factor model of the GHQ-12**.

* P < 0.01

(ii) The indices were improved with a model that split the items into positive versus negative. CFA yielded a 10-item two-factor model (positive vs. negative items) that fitted the data very well. The figures for the indices were: Goodness of Fit Index = 0.93, Adjusted Goodness of Fit Index = 0.90, Root Mean Square Error of Approximation (RMSEA) = 0.02, and χ^2^/df = 2.11.

(iii) Finally, we tested the three factors identified by Graetz ("anxiety and depression", "social dysfunction" and "loss of confidence") [15]. Analysis showed that the model was highly consistent with our data. CFA yielded a 12-item three-factor model that fitted the data very well: Goodness of Fit Index = 0.93, Adjusted Goodness of Fit Index = 0.90, Root Mean Square Error of Approximation (RMSEA) = 0.03, and χ^2^/df = 2.26 (Table 1 and Figure 1). In summary, the two and three factor models fitted the data very well, while the one factor model did not.

The internal consistency of the questionnaire was measured using Cronbach's alpha coefficient. This coefficient was found to be 0.78 for the uni-dimensional model, while for the two-factor and three-factor models the alpha values were found to be: Anxiety/depression, 0.84; Social dysfunction, 0.76; and Loss of confidence, 0.81.

#### 2. The Subjective Vitality Scale (VS)

The CFA yielded a six-item uni-dimensional model that fitted the data well. The following indices were found: Goodness of Fit Index = 0.90, Adjusted Goodness of Fit Index = 0.89, Root Mean Square Error of Approximation (RMSEA) = 0.2, and χ^2^/df = 11.01 (Table 1).

The internal consistency of the questionnaire was measured using Cronbach's alpha coefficient and was found to be 0.83, well above the threshold for a satisfactory value.

### Relationship between the GHQ-12 and the VS

The correlation between the GHQ-12 and the VS scores was investigated and as expected a significant negative correlation emerged (*r *= -0.71, P < 0.01), indicating that those who were more distressed showed lower levels of subjective vitality (Table 2).

**Table 2:** Descriptive statistics and correlations between the GHQ-12 and the VS (n = 217)

			**Correlations**				
	**Mean**	**SD**	**1**	**2**	**3**	**4**	**5**

**1. The VS score**	22.4	7.4	1.00				

**2. GHQ-12, anxiety and depression**	7.8	6.7	-0.10	1.00			

**3. GHQ-12, social dysfunction**	3.7	4.50	-0.08	0.08	1.00		

**4. GH-12, loss of confidence**	5.9	2.2	-0.01	0.02	-0.09	1.00	

**5. Total score of the GHQ-12**	17.4	8.0	-0.71*	0.79*	0.49*	0.26*	1.00

* Significant at < 0.01 level.

## Discussion

The GHQ is a well-known instrument for measuring minor psychological distress and has been translated into a variety of languages [2-6,16,17]. However, it is not a tool for indicating a specific diagnosis. This paper reports data from a validation study of the 12-item GHQ in France. In general, the findings showed satisfactory results and were comparable with most research findings throughout the world [18-21]. In addition, we report the first data from France on the Subjective Vitality Scale (VS), lending support to its validity for use in French populations. Cronbach's alpha in our study was 0.83, very close to the value found by Bostic et al. [10]; for their two data sets, they reported Cronbach's alpha values of 0.80 and 0.89.

Although the GHQ-12 was originally developed as a unitary screening measure for psychological problems, there have been efforts to determine whether it has a multidimensional structure [22]. The World Health Organization study of psychological disorders in general health care in 15 different centres indicated that there is substantial factor variation between centres for the GHQ-12 [23]. However, our results showed not only that two factors expressing anxiety/depression and social dysfunction could be identified, but also that three factors (i.e. anxiety/depression, social dysfunction and loss of confidence) are evident. The findings from the present study showed that the French version of the GHQ-12 is a valid measure of psychological distress, but the questionnaire has a different factor structure from that in the original language.

Since there was a strong correlation between the GHQ-12 and the VS, the finding also lend further support to the notion that vitality is both experientially important and meaningful, and contains physical and psychological determinants [7]. In addition, since the existence of links between vitality and a number of health conditions ranging from sleep difficulties to somatic illnesses has been well reviewed [11], use of the VS is recommended in future studies. However, none of the GHQ-12 subscales were correlated with the VS score. This implies that in practice one should avoid correlating vitality with anxiety and depression, social dysfunction or loss of confidence alone.

In general, the findings from this study indicated that there is relatively little mental illness in old people practising physical activities in France and this is strongly associated with their perceived vitality. However, it should be noted that our participants were a selected sample, so these findings cannot be generalized to the whole elderly population of France.

## Conclusion

The findings suggest that the French version of the GHQ-12 is a reliable and valid instrument for measuring minor psychological distress in old people and has a good factor structure. In addition, this is the first study to test the reliability and factor structure of the Subjective Vitality Scale (VS) in France. The results show that it has good psychometric properties in terms of internal consistency and factor structure. Finally, as expected, the relationship between the two instruments was significantly negative, lending support to their convergent validity.

## Competing interests

The authors declare that they have no competing interests.

## Authors' contributions

MSY was the main investigator and analysed the data and wrote the first draft. AI and CR contributed to the study design and the analysis. AM contributed to the analysis and wrote the final manuscript. All authors read and approved the final manuscript.
